# Landscape extraordinariness and visitors' psychological restoration in national park settings: a chain mediation model of awe, construal level, and connectedness to nature

**DOI:** 10.3389/fpsyg.2026.1887097

**Published:** 2026-07-27

**Authors:** Zhiyue Zhu, Jinhe Zhang, Xiaoxin Chen, Anzhi Li, Han Song

**Affiliations:** 1 School of Geography and Ocean Science, Nanjing University, Nanjing, China; 2 Huangshan Park Ecosystem Observation and Research Station, Ministry of Education, Huangshan, China; 3 Academy of Plateau Science and Sustainability, Xining, China

**Keywords:** awe, connectedness to nature, construal level, extraordinary nature, landscape extraordinariness, national parks, psychological restorative benefits

## Abstract

**Introduction:**

The psychological restorative potential of ordinary green spaces is well documented, yet the mechanisms linking extraordinary natural landscapes to restoration remain underexplored. This study conceptualizes landscape extraordinariness as a continuous subjective perceptual construct and proposes a chain mediation model, grounded in the Cognitive-Affective Personality System framework, to examine the roles of awe, construal level, and connectedness to nature in visitors' psychological restorative benefits in national park settings.

**Methods:**

Structured questionnaires were administered to 457 visitors at Sanjiangyuan National Park and Qinghai Lake National Park (candidate area), China. Confirmatory factor analysis assessed measurement quality, and structural equation modeling with bootstrap resampling using 5,000 samples and 95% bias-corrected confidence intervals tested the hypothesized relationships.

**Results:**

Landscape extraordinariness was positively associated with awe, connectedness to nature, and psychological restorative benefits, but was not directly associated with construal level. Awe was positively associated with higher construal level and stronger connectedness to nature, while connectedness to nature was positively associated with psychological restorative benefits. Bootstrap analyses supported the proposed chain mediation model, with significant indirect associations involving awe and connectedness to nature and an additional contribution of construal level within the longer sequential process. Overall, the findings supported partial mediation.

**Discussion:**

These findings clarify the affective-cognitive mechanisms associated with psychological restoration in extraordinary landscapes and underscore the role of awe in linking extraordinary landscape perception with higher-level construal. The results extend extraordinary nature research to authentic national park contexts and provide practical implications for visitor experience design and nature-based wellbeing interventions.

## 1 Introduction

Growing recognition of global mental health challenges has elevated interest in nature-based interventions as accessible, low-cost means of promoting psychological wellbeing. The World Health Organization estimates that approximately 1 in 8 people worldwide lives with a mental disorder, yet mental health expenditures remain persistently low, with most countries allocating a median of around 2% of total health budgets to mental health services; the majority of affected individuals receive no effective intervention (World Health Organization, 2022, 2025). Against this backdrop, natural environments have attracted considerable scholarly attention as widely accessible psychological health resources, and the question of how contact with nature is associated with mental wellbeing has become a central concern across disciplines.

Two theoretical frameworks have long provided systematic explanations for the restorative potential of nature. Attention Restoration Theory (ART) proposes that natural environments facilitate the recovery of directed attentional fatigue through qualities such as soft fascination, a sense of being away, and extent (Kaplan and Kaplan, 1989). Stress Recovery Theory (SRT) emphasizes that natural scenes activate the parasympathetic nervous system and rapidly alleviate physiological stress responses (Ulrich et al., 1991). Both frameworks have been extensively validated: experimental studies demonstrate that walking in natural settings not only restores attention and suppresses maladaptive rumination (Bratman et al., 2015) but also reduces activation in stress-related brain regions (Sudimac et al., 2022), while systematic reviews and meta-analyses confirm measurable benefits of nature contact across emotional, cognitive, and physiological domains (Coventry et al., 2021; Ohly et al., 2016).

Notwithstanding this body of evidence, both theoretical frameworks and their supporting empirical literature have been developed predominantly in relation to “ordinary nature”—urban parks, community green spaces, and common forests—while natural landscapes characterized by vast scale, visual grandeur, and high ecological rarity have remained largely outside the scope of systematic inquiry. Joye and Bolderdijk (2015) were the first to conceptualize such settings as “extraordinary nature” (e.g., canyons, glaciers), demonstrating experimentally that exposure to extraordinary nature produces stronger effects on mood and prosocial behavior than ordinary nature, with awe identified as the central psychological mechanism. Subsequent research employing electroencephalography, wilderness fieldwork, and virtual reality has confirmed the positive contributions of extraordinary nature to psychological restoration and self-transcendence (Bai et al., 2025; Hao et al., 2024; Løvoll and Sæther, 2022).

Despite widespread recognition of extraordinary nature's therapeutic potential, two limitations persist in the existing literature. At the measurement level, prior research has predominantly treated “extraordinary nature” as a holistic environmental category, comparing its effects with those of ordinary nature, without translating specific landscape features into operationalizable subjective assessment variables. This approach renders the psychological mechanisms of extraordinary nature somewhat opaque. At the level of research context, most prior studies have relied on laboratory-based visual stimuli (Bai et al., 2025). Although prior research distinguishes positive and threatening forms of awe, the conditions under which extraordinary landscapes are associated with restorative experiences remain insufficiently understood (Liu et al., 2023). National parks provide a relevant field setting for examining visitors' experiences of large-scale, distinctive, and ecologically rare landscapes.

Against this background, the present study conceptualizes landscape extraordinariness as a continuous individual-level perceptual construct capturing visitors' overall appraisal of a landscape's grandeur, remoteness, mystery, complexity, and uniqueness. Unlike the binary categorization of extraordinary vs. ordinary nature, landscape extraordinariness varies according to the perceived intensity of landscape attributes that depart from everyday experience. The study was conducted in Sanjiangyuan National Park and Qinghai Lake National Park (candidate area) in China. Theoretically, CAPS serves as the overarching organizing framework, hot/cool system theory informs the proposed affective–cognitive ordering, and Construal Level Theory clarifies the proposed association between awe and higher-level construal.

This study makes three primary contributions. First, it operationalizes landscape extraordinariness as a continuous subjective perception variable rather than treating extraordinary nature as a binary environmental category. This approach enables a more fine-grained examination of how different degrees of perceived extraordinariness are associated with visitors' psychological responses. Second, by integrating CAPS theory, hot/cool system theory, and CLT, the study proposes and tests an affective–cognitive sequence in which awe is associated with higher-level construal. This highlights the cognitive processing differences associated with awe rather than treating awe as an isolated emotional response. Third, the study positions connectedness to nature as a proximal mediator linking affective and cognitive responses to psychological restorative benefits. In doing so, the study examines not only whether landscape extraordinariness is associated with restoration, but also the psychological pathways through which such benefits may emerge in national park settings.

## 2 Research hypotheses

### 2.1 Theoretical framework

The present study draws on the Cognitive-Affective Personality System (CAPS) theory as an overarching framework for explaining how perceived landscape extraordinariness is translated into psychological restorative benefits. CAPS proposes that individuals' psychological and behavioral responses arise from the dynamic operation of cognitive and affective processing units activated within a particular context (Mischel and Shoda, 1995). The framework has been applied across organizational behavior, organizational psychology, and tourism research, demonstrating its value in explaining how situational cues activate internal psychological processes and shape subsequent responses (Wang et al., 2018; Frieder et al., 2018; Heslin et al., 2019). For example, Wang et al. (2024) employed CAPS to examine how environmental atmosphere in a virtual festival context influences visitors' evaluations and behavioral responses through cognitive and affective processing. This application illustrates the relevance of CAPS to tourism settings in which salient experiential cues shape visitors' internal states and subsequent responses.

Extending this general situation–processing–response logic to extraordinary natural landscapes, the present study focuses on the situational activation of cognitive and affective processing units. Perceived landscape extraordinariness is conceptualized as a salient situational cue, awe as the affective processing unit, and construal level as the cognitive processing unit. Connectedness to nature is subsequently positioned as the proximal psychological mechanism through which these internal responses are associated with psychological restorative benefits. Thus, CAPS serves primarily to organize the principal situational, affective, and cognitive components of the proposed model.

To further clarify the relationship between affective and cognitive processing, the study draws on hot/cool system theory. The hot system governs immediate emotional responses to salient and high-arousal stimuli, whereas the cool system supports more abstract and reflective cognitive processing (Metcalfe and Mischel, 1999). Extraordinary landscapes characterized by vastness, novelty, rarity, and sensory intensity are therefore theorized to elicit awe as a hot-system response, which may facilitate subsequent cool-system processing in the form of higher-level construal. Construal Level Theory further clarifies this cognitive shift: the perceived vastness and need for accommodation associated with awe may increase psychological distance and promote more abstract mental representations (Keltner and Haidt, 2003; Shiota et al., 2007; Trope and Liberman, 2010). Higher-level construal may then enable visitors to move beyond concrete landscape features and develop a more holistic understanding of the human–nature relationship, thereby strengthening connectedness to nature and contributing to psychological restorative benefits. Accordingly, CAPS provides the overarching organizing framework, hot/cool system theory specifies the proposed affective–cognitive ordering, and Construal Level Theory clarifies the nature of the cognitive shift.

### 2.2 Landscape extraordinariness

Joye and Bolderdijk (2015) introduced the concept of “extraordinary nature” to describe visually striking natural phenomena—such as volcanoes, glaciers, and mountain canyons—capable of inducing wonder, demonstrating that these environments produce stronger emotional and prosocial effects than ordinary nature. Unlike ordinary natural settings that operate through the low-arousal attentional restoration mechanism described by ART, extraordinary nature influences individuals primarily through high-arousal emotional activation, with awe and stimulation identified as the two primary mediating emotions (Herzog et al., 1997; Quesnel and Riecke, 2018). Subsequent research employing electroencephalography, wilderness fieldwork, and virtual reality has confirmed the restorative and self-transcendent effects of extraordinary nature across multiple contexts (Bai et al., 2025; Hao et al., 2024; Løvoll and Sæther, 2022).

Notwithstanding this body of evidence, existing research has consistently treated “extraordinary nature” as a binary environmental category, comparing its effects with ordinary nature without translating its defining landscape features into an operationalizable continuous individual-level variable. This approach renders the psychological mechanisms of extraordinary nature difficult to investigate precisely. The present study therefore introduces “landscape extraordinariness” as a subjective perceptual construct representing the core landscape attributes that distinguish extraordinary from ordinary nature. Unlike the categorical binary classification, landscape extraordinariness is operationalized as a continuous variable that varies with individual perceptual sensitivity across five evaluative indicators: grandeur, remoteness, mystery, complexity, and uniqueness. Higher scores reflect stronger perception of landscape features that transcend everyday experience.

Several theoretical mechanisms support a direct effect of landscape extraordinariness on connectedness to nature. Van Rompay et al. (2023) demonstrated that expansive natural spaces strengthen individuals' awareness of their own finitude through the proportional relationship between body and environment, weakening self-centered cognitive modes and facilitating connectedness to nature. Løvoll and Sæther (2022) similarly found in an Arctic wilderness field study that visitors immersed in grand landscapes more readily perceive themselves as part of the natural world and the cycle of life. Together, these studies suggest that perceiving a landscape as expansive and distinctive may be associated with a stronger sense of belonging to the natural world. Based on this reasoning, the following hypothesis is proposed:

H1: Landscape extraordinariness has a positive and significant effect on connectedness to nature.

Keltner and Haidt's (2003) prototype model specifies that awe is constituted by two core elements: the perception of vastness and a need for accommodation arising when existing cognitive schemas cannot integrate the current experience. Shiota et al. (2007) further identified complexity and rarity as the prototypical attributes that trigger such schema incompatibility. Extraordinary natural landscapes fulfill both conditions: their physical scale and spatial extent directly trigger perceptions of vastness, while their rarity and complexity disrupt habitual cognitive frameworks. Experimental studies using virtual images of the Himalayas and the Grand Canyon have confirmed that such scenes reliably elicit awe (Quesnel and Riecke, 2018), and tourism research identifies scenic grandeur and expansiveness as the primary sensory cues for awe in natural settings (Xu and Hu, 2023). Accordingly:

H2a: Landscape extraordinariness has a positive and significant effect on awe.

### 2.3 Awe

Awe is a complex, self-transcendent emotion arising from the encounter with something vast that challenges and potentially expands one's existing mental frameworks (Keltner and Haidt, 2003). In nature-based contexts, awe has been positioned as one of the most influential pathways from environmental perception to psychological transformation, linked to reduced self-focus, expanded social identity, and enhanced wellbeing across diverse cultures and settings (Piff et al., 2015; Yang et al., 2018).

The mechanism linking awe to connectedness to nature can be understood through resource allocation theory. Self-focused attention consumes limited cognitive resources, thereby reducing awareness of the external natural environment (Frantz et al., 2005). Awe disrupts this self-centered allocation pattern and redirects attention outward through two primary pathways. First, awe induces the “small self” experience—a diminishment of self-concept that enables individuals to perceive themselves as part of a greater natural whole rather than as a separate, bounded entity (Piff et al., 2015; Takano and Nomura, 2021). Second, awe enhances openness and dissolves the psychological boundary between the self and the external world; boundary dissolution is recognized as a central prerequisite for the formation of connectedness to nature (Lee et al., 2015; Mayer and Frantz, 2004). Empirical support for these mechanisms is robust: Piff et al.'s (2015) field experiment in Yosemite National Park found that participants who experienced awe drew significantly smaller self-images in subsequent tasks, and daily diary tracking confirmed that awe was persistently associated with lower levels of self-focus. Studies using high-awe landscape stimuli similarly observed reduced self-boundary perception and enhanced environmental connectedness among participants (Röhr, 2022). Accordingly:

H2b: Awe has a positive and significant effect on connectedness to nature.

Integrating the CAPS framework, landscape extraordinariness as an external situational stimulus activates awe as an affective processing unit that, in turn, acts on connectedness to nature. This transmission pathway has been confirmed in tourism contexts: visitors in expansive natural settings frequently experience intense awe (Yang et al., 2018), and this affective state subsequently enhances their perceived connectedness to nature (Lengieza and Swim, 2021). Importantly, connectedness to nature is not merely an attitudinal end state but functions as the proximal psychological mechanism through which nature contact generates restorative outcomes. The biophilia hypothesis holds that when the innate human need for nature affiliation is satisfied, individuals derive significant psychological restorative benefits (Wilson, 1984). Mayer et al. (2009) experimentally demonstrated that the direct effect of nature exposure on psychological restoration disappeared entirely once connectedness to nature was controlled for, confirming its role as a necessary mediating pathway. The affective chain identified above—whereby landscape extraordinariness elicits awe, and awe deepens connectedness to nature—is therefore expected to extend through connectedness to nature to psychological restorative benefits as the ultimate outcome. The following hypothesis is therefore proposed:

H2: Awe and connectedness to nature sequentially mediate the relationship between landscape extraordinariness and psychological restorative benefits.

### 2.4 Construal level

Construal Level Theory (CLT) proposes that individuals' responses to events are shaped by the degree of abstraction in their mental representations, defined as construal level (Trope and Liberman, 2010). High construal level corresponds to abstract, decontextualized representations focused on essential features, while low construal level corresponds to concrete, context-bound representations emphasizing surface details. The theory has been extended from social psychology to environmental psychology and tourism research, where it has been applied to analyze pro-environmental behavior (Chen, 2020; Reczek et al., 2018), visitor behavioral intentions, and destination choice (Craig et al., 2021; Li and Ma, 2024; Li et al., 2019). While early scholarship treated construal level as a stable trait (Vallacher and Wegner, 1989), the field has increasingly recognized it as a state variable susceptible to situational manipulation (Wang et al., 2019; Li et al., 2020). The present study adopts this state-level perspective, treating construal level as a situational cognitive variable responsive to landscape stimuli.

The potential influence of landscape extraordinariness on construal level operates through psychological distance mechanisms. ART identifies “extent”—a sense of integration with the environment in which the world feels larger than oneself—as one of the key restorative qualities of natural experience (Kaplan, 1995), while SRT specifies that the expansiveness of natural scenes influences both immediate physiological responses and subsequent cognitive appraisal processes (Ulrich, 1993). The large scale of extraordinary natural landscapes may be relevant to the spatial dimension of psychological distance. According to CLT, greater psychological distance is associated with more abstract construal (Adler and Sarstedt, 2021). Experimental research has also shown that spatially distant consequences can be processed at a more abstract level (Chu, 2022). The landscapes in the study settings therefore provide a relevant context for examining the proposed association between landscape extraordinariness and construal level. Accordingly:

H3a: Landscape extraordinariness is significantly associated with visitors' adoption of a higher level of construal.

When individuals operate at high construal levels, their mental representations shift toward holistic and abstract orientations (Septianto et al., 2023). Connectedness to nature requires exactly this capacity to transcend concrete sensory experience—it entails a holistic understanding of the human–nature relationship and an intrinsic recognition of the value of the community of life. High construal level additionally directs attention toward desirability-related value information rather than concrete situational details (Eyal et al., 2009; Fujita et al., 2008); in natural settings, this means a shift of focus from surface sensory stimuli toward the deeper meaning associated with one's relationship with nature. Xu and Hu (2023) confirmed this pathway in a tourism context, demonstrating that state construal level is associated with connectedness to nature and strengthens environmental responsibility intentions via the small-self experience. The following hypothesis is therefore proposed:

H3b: Higher-level construal is significantly associated with stronger connectedness to nature.

Within the CAPS framework, landscape extraordinariness can act on connectedness to nature through the activation of construal level as a cognitive processing unit. However, the core premise of hot/cool system theory—that high-arousal contexts prioritize hot-system activation—implies that the affective and cognitive pathways may not be entirely independent but may involve a sequential activation order. Whether landscape extraordinariness can exert an independent cognitive mediation effect absent prior affective activation remains an open empirical question. This study includes the independent cognitive mediation pathway in its analytical model to evaluate its explanatory power directly. Following the same reasoning that connectedness to nature constitutes the indirect pathway through which nature-related psychological states translate into restorative benefits (Wilson, 1984; Mayer et al., 2009), the cognitive mediation chain—in which landscape extraordinariness is associated with a higher level of construal, and higher-level construal is associated with stronger connectedness to nature—is likewise expected to extend to psychological restorative benefits:

H3: Construal level and connectedness to nature sequentially mediate the relationship between landscape extraordinariness and psychological restorative benefits.

### 2.5 Chain mediation of awe and construal level

A direct relational mechanism links awe to construal level. One of the core dimensions of the awe experience is the “small self”—a profound perceptual shrinkage of the individual self—which prompts individuals to adopt a global rather than egocentric perspective in understanding the surrounding world (Piff et al., 2015). Vast natural phenomena, as paradigmatic awe elicitors, inherently require perception from a distanced vantage point, implying greater psychological distance from the self (Keltner and Haidt, 2003; Shiota et al., 2007). According to CLT, psychological distance and mental abstraction are bidirectionally associated such that greater distance corresponds to higher-order construal (Trope and Liberman, 2010); the sense of smallness and distancing induced by awe therefore naturally orients individuals toward more abstract cognitive processing. Furthermore, the need for accommodation triggered by awe—requiring individuals to update their cognitive schemas to integrate an experience that exceeds their current mental frameworks (Shiota et al., 2007)—reflects precisely the higher-level schematic processing that characterizes high construal (Bullard et al., 2019). Xu and Hu (2023) confirmed this link in a tourism context, demonstrating that awe elevates visitors' state construal level, enabling more abstract processing of environmental stimuli. Li and Su (2024) further demonstrated that construal level serves as a core mediating mechanism in the pathway from awe to subsequent attitudinal and behavioral outcomes.

Integrating these insights with hot/cool system theory, the extraordinary landscape preferentially activates awe as a hot-system affective unit; the need for cognitive accommodation induced by awe then drives the cool system into a high-order construal mode; and this abstract self–nature cognition ultimately facilitates the formation of connectedness to nature. Given that the complete affective–cognitive chain culminates in connectedness to nature—which serves as the proximal psychological pathway to restoration (Mayer et al., 2009; Wyles et al., 2019)—this chain is expected to extend beyond connectedness to psychological restorative benefits as the distal outcome. The following hypothesis is therefore proposed:

H4a: Awe is significantly associated with visitors' adoption of a higher level of construal.

H4b: Awe, construal level, and connectedness to nature chain-mediate the relationship between landscape extraordinariness and psychological restorative benefits.

### 2.6 Connectedness to nature and psychological restorative benefits

The biophilia hypothesis holds that humans have an evolved innate need to affiliate with the natural world, and that when this need is satisfied, individuals derive significant psychological and physical restorative benefits (Wilson, 1984). Empirical research has consistently confirmed that connectedness to nature is positively associated with multiple dimensions of psychological wellbeing, including personal growth, self-acceptance, and life satisfaction (Nisbet et al., 2011). Wyles et al. (2019) reported a positive association between connectedness to nature and psychological restoration. Mayer et al. (2009) experimental work provides particularly instructive evidence: when connectedness to nature was incorporated into the analytical model, the direct effect of walking location on psychological restoration disappeared entirely, indicating that connectedness to nature constitutes the underlying pathway through which natural environments generate restorative benefits. The following hypothesis is therefore proposed:

H5: Connectedness to nature has a positive and significant effect on psychological restorative benefits.

Evidence for a direct effect of landscape extraordinariness on psychological restorative benefits comes from multiple converging lines of research. Awe-inspiring landscapes have been shown to provide stronger attentional restoration and mood enhancement compared to ordinary natural environments, and studies using grand natural imagery as experimental stimuli confirm that such scenes improve psychological restoration by enhancing attentional performance and subjective restoration perception (Collado and Manrique, 2020). Over longer timeframes, Bogaert et al. (2024) week-long intervention study found that high-extraordinariness natural landscapes reduced rumination and enhanced subjective wellbeing, indicating sustained psychological health benefits beyond the immediate experience. Accordingly:

H6: Landscape extraordinariness has a positive and significant effect on psychological restorative benefits.

### 2.7 Theoretical model

**Figure 1:**
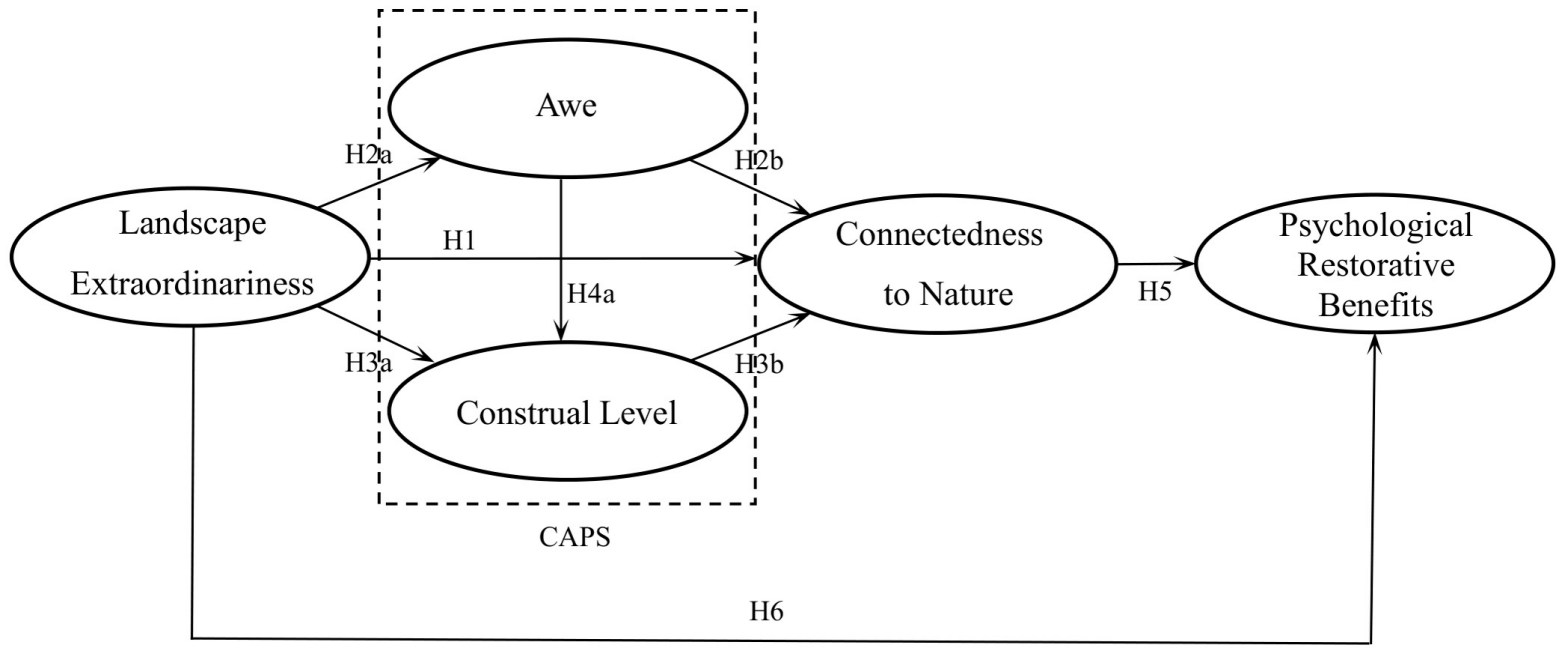
Theoretical model.

Based on the theoretical arguments and empirical evidence reviewed above, the present study proposes the theoretical model presented in Figure 1.

## 3 Materials and methods

### 3.1 Study area

This study selected Sanjiangyuan National Park and Qinghai Lake National Park (candidate area) as the research settings. Sanjiangyuan National Park is among the first cohort of officially designated national parks in China. Qinghai Lake was approved for national park creation by the National Forestry and Grassland Administration in June 2022 and completed all establishment requirements in March 2024, entering a national-level effectiveness evaluation phase; its ecological conservation management system, visitor infrastructure, and safety governance mechanism have been fully constructed in accordance with national standards such as the National Park Establishment Norms (National Forestry, and Grassland Administration, 2022), and the site has already been used as a research setting for national park visitor behavior studies (Wang et al., 2025). Both parks are situated in the core zone of the Qinghai-Tibet Plateau and share high-altitude terrain, large-scale physical landscapes, and low anthropogenic disturbance. These characteristics make the two sites suitable for examining visitors' perceptions of landscape extraordinariness and their associations with psychological responses.

### 3.2 Measures

All constructs were measured using multiple-item scales adapted from established instruments in prior research. Landscape extraordinariness was measured using a five-item semantic differential measure adapted from Hao et al. (2024). In the present study, landscape extraordinariness is conceptualized as a unidimensional reflective latent variable representing visitors' overall subjective appraisal of the degree to which a landscape departs from ordinary natural settings (Jarvis et al., 2003). Five semantic-differential items assessing grandeur, remoteness, mystery, complexity, and uniqueness served as reflective indicators of this overall appraisal. The indicators were expected to covary as manifestations of the same underlying perception and were not treated as separate components whose combination forms the construct. The original “awe” item label used by Hao et al. (2024) was refined to “grandeur” to focus specifically on the perceived physical scale of the landscape. Items were scored on a five-point bipolar format, and respondents indicated their position on each continuum based on their overall impression of the landscape. All items were contextually adapted for the national park setting.

All remaining constructs employed five-point Likert scales (1 = strongly disagree, 5 = strongly agree). Awe was measured using six items adapted from the nature-awe scales developed by Bai et al. (2025) and Collado and Manrique (2020). Construal level was assessed with three items adapted from the state construal level scales of Burgoon et al. (2013) and Wang et al. (2019), capturing the degree of abstractness in visitors' cognitive representations under situational stimulation. Connectedness to nature was measured with four items adapted from Gosling and Williams (2010). Psychological restorative benefits were assessed using seven items adapted from the perceived restoration evaluation scale of Luo et al. (2022).

To enhance the contextual applicability of the adapted scales to the extraordinary natural landscape setting, three subject-matter experts in the field were invited to review all items for content accuracy and linguistic appropriateness; their suggestions were incorporated into the final revisions.

### 3.3 Pilot study

To verify the applicability of the adapted scales in the national park context, a pilot study was conducted via an online survey platform (Wenjuanxing), recruiting 120 participants who had visited a national-level protected area within the preceding 3 years. Participants completed all measurement items and rated each item's clarity on a five-point scale (1 = not at all clear, 5 = very clear). Based on participant feedback, minor wording revisions were made to improve the clarity of several technical terms. Results indicated that Cronbach's α coefficients for all scales exceeded 0.80, mean item clarity was 4.4 (*SD* = 0.3), and exploratory factor analysis (EFA) confirmed that all items loaded onto their intended constructs with no evidence of cross-factor loading. All items were retained for the main survey.

### 3.4 Data collection

Data were collected between 15 and 25 July 2025. The research team conducted mobile sampling along a route spanning Xining–Yushu–Zaduo–Zhiduo–Gonghe, administering face-to-face questionnaires at representative viewpoints including the Yangtze Source Park entrance monument, the Bayan Har Mountain pass, and the Angsai Grand Canyon within the Sanjiangyuan park zone, and at the Erlangjian core viewing area of Qinghai Lake. Given the substantial differences in visitor accessibility and traffic volume between the two sites—Qinghai Lake receives considerably more visitors as an established high-plateau tourism node, while the Sanjiangyuan core zone attracts fewer visitors due to its remote location—a composite sampling pool approach was adopted. Questionnaires were distributed using a convenience sampling procedure: trained researchers with backgrounds in tourism geography intercepted visitors at each viewpoint and provided necessary semantic guidance during questionnaire completion to ensure data quality in the high-altitude field environment.

A total of 498 questionnaires were collected. After excluding responses with excessively short completion times, obvious patterned responding, or missing values on key items, 457 valid questionnaires were retained, yielding an effective response rate of 91.77% (Sanjiangyuan sub-sample: *N* = 154; Qinghai Lake sub-sample: *N* = 303). This sample size substantially exceeds Stevens' recommended minimum of 15 observations per measured item for structural equation modeling (Pituch and Stevens, 2016). Before merging the two sub-samples, additional comparability checks were conducted to address the unequal group sizes between Sanjiangyuan National Park and Qinghai Lake National Park. Welch's independent-samples *t*-tests were used to compare the five core construct scores because this procedure is more robust to unequal group sizes and potential variance heterogeneity than conventional independent-samples *t*-tests. Mann–Whitney U tests were further conducted as non-parametric sensitivity checks. In addition, multi-group CFA was performed to examine whether the measurement structure was stable across the two site-based sub-samples. Based on these checks, reported in the Results section, the two sub-samples were merged for all subsequent analyses (*N* = 457).

The socio-demographic characteristics of the sample are presented in Table 1. Male respondents accounted for 42.7% of the sample and female respondents for 57.3%. The 18–25 age group was the most represented (37.2%), followed by the 26–35 group (25.8%) and the 36–45 group (20.8%), indicating a predominantly young-to-middle-aged visitor profile. Respondents with a bachelor's degree constituted the largest education group (47.5%), and those with a master's degree or above accounted for 18.8%, reflecting a generally high level of educational attainment. In terms of occupation, corporate or enterprise employees (22.1%) and students (19.9%) were the two largest groups. The most common income bracket was ¥50,000–1,00,000 per annum (44.6%). Respondents came from 28 provincial-level administrative regions, with Guangdong, Shaanxi, and Sichuan being the most represented provinces (8.8%, 8.8%, and 8.3%, respectively), indicating a geographically broad sample with reasonable representativeness.

**Table 1:** Demographic characteristics of the sample (*N* = 457).

Demographic traits		*n*	%
**Gender**	Male	195	42.70%
	Female	262	57.30%
**Age**	18–25	170	37.20%
	26–35	118	25.80%
	36–45	95	20.80%
	46–55	43	9.40%
	Above 55	31	6.80%
**Education**	High school or below	70	15.40%
	Associate's degree	84	18.30%
	Bachelor's degree	217	47.50%
	Master's degree or above	86	18.80%
**Occupation**	Government/public sector employee	28	6.10%
	Corporate/enterprise employee	101	22.10%
	Self-employed/entrepreneur	78	17.10%
	Manual worker	20	4.40%
	Farmer	15	3.30%
	Military personnel	4	0.90%
	Student	91	19.90%
	Professional/technical worker (e.g. teacher, doctor)	78	17.10%
	Retired	26	5.70%
	Other	16	3.50%
**Annual personal income**	Below ¥ 50,000	84	18.40%
	¥50,000–100,000	204	44.60%
	¥100,000–200,000	98	21.40%
	Above ¥200,000	71	15.50%

### 3.5 Data analysis

Prior to the main measurement and structural analyses, site-subsample comparability was assessed using Welch's *t*-tests, Mann–Whitney U tests, and multi-group CFA to evaluate whether the Sanjiangyuan and Qinghai Lake subsamples could be appropriately pooled. Statistical analyses were conducted using SPSS 26.0 and Amos 26.0. The analytical procedure comprised four sequential steps. First, Harman's single-factor test and the unmeasured latent method construct (ULMC) approach were employed to evaluate the risk of common method bias (CMB). Second, confirmatory factor analysis (CFA) was conducted to assess the reliability and validity of the measurement model. Third, structural equation modeling (SEM) was used to test the hypothesized direct path relationships. To evaluate whether the hypothesized ordering among mediators fit the data better than plausible alternative configurations, two competing structural models were estimated and compared against the proposed model using the Akaike Information Criterion (AIC) and other fit indices (Kline, 2016). Fourth, the bootstrap resampling procedure (5,000 iterations, 95% bias-corrected confidence interval) was applied to examine the significance of the complete indirect effects from landscape extraordinariness to psychological restorative benefits through the affective mediation path, the independent cognitive mediation path, and the chain mediation path (Preacher and Hayes, 2008).

## 4 Results

### 4.1 Common method bias assessment

Because all variables were self-reported by the same respondents, the potential for common method bias (CMB) was addressed through both procedural and statistical controls. At the procedural level, the landscape extraordinariness scale adopted a semantic differential format while all other scales used a Likert-type format; this deliberate design contrast was intended to interrupt habitual response patterns, a recognized method for reducing common method variance. Statistically, Harman's single-factor test showed that the largest unrotated component accounted for 30.105% of total variance, falling below the 50% threshold (Podsakoff et al., 2003). To apply a more rigorous check in line with the recommendation by Kock et al. (2021) for tourism research, an unmeasured latent method construct (ULMC) model was further estimated. The extended model including a common method factor produced fit indices of χ^2^/*df* = 3.013, CFI = 0.908, TLI = 0.896, and RMSEA = 0.066. The CFI change relative to the baseline model was ΔCFI = 0.004, below the 0.01 cut-off (Cheung and Rensvold, 2002); all observed-variable path coefficients shifted by less than 0.2, and the original model's path relationships and significance levels were substantively unchanged. These results collectively indicate that serious CMB was absent.

### 4.2 Measurement model

The overall fit of the measurement model was acceptable: χ^2^/*df* = 3.096, RMSEA = 0.077, IFI = 0.905, TLI = 0.892, and CFI = 0.904. Reliability was satisfactory across all constructs: Cronbach's α coefficients ranged from 0.779 to 0.892, each surpassing the 0.70 lower bound recommended by Nunnally (1978), and composite reliability (CR) values ranged from 0.778 to 0.894, meeting the standard of Hair et al. (2019).

For convergent validity, standardized factor loadings ranged from 0.513 to 0.862—the majority exceeding 0.70—and AVE values ranged from 0.476 to 0.565. The AVE for connectedness to nature (0.476) was marginally below the conventional 0.50 benchmark; however, Fornell and Larcker (1981) note that when the corresponding CR exceeds 0.60, an AVE above 0.40 remains acceptable. The CR for this construct was 0.784, so its convergent validity was regarded as sufficient. Full measurement statistics are presented in Table 2.

**Table 2:** Reliability and validity of the measurement model.

Latent variable	Item	Factor loading	Cronbach's α	AVE	CR
Landscape extraordinariness (LE)	Small and ordinary ↔ Grand and magnificent	0.803	0.860	0.557	0.861
	Close to everyday environments ↔ Remote from the mundane world	0.673			
	Obvious and transparent ↔ Mysterious and enigmatic	0.627			
	Monotonous and dull ↔ Rich and diverse	0.803			
	Common and ubiquitous ↔ Unique and one-of-a-kind	0.805			
Awe (AW)	The landscape here inspires a deep sense of awe in my heart.	0.856	0.881	0.565	0.885
	The landscape here feels sacred and solemn.	0.552			
	The beauty of this landscape cannot be fully expressed in words.	0.788			
	The landscape here fills me with awe.	0.769			
	The landscape here gives me a feeling of surpassing the ordinary.	0.777			
	The landscape here touches me deeply.	0.733			
Construal level (CL)	While appreciating the landscape here, my thoughts are more (concrete vs. abstract)	0.717	0.779	0.540	0.778
	While appreciating the landscape here, I tend to focus on the (details vs. big picture)	0.700			
	While appreciating the landscape here, I tend to focus on the (specifics vs. overall feeling)	0.785			
Connectedness to nature (CN)	Here I feel I am part of the natural world	0.689	0.784	0.476	0.784
	Here I feel connected with the natural world around me.	0.687			
	Here I experience a sense of oneness with the natural environment around me.	0.684			
	Here I feel that my own welfare is linked to the welfare of the natural world.	0.700			
Psychological restorative benefits (PRB)	Appreciating the landscape here makes me feel restored.	0.755	0.892	0.552	0.894
	Appreciating the landscape here makes me forget everyday worries.	0.806			
	Appreciating the landscape here gives me a break from my day-to-day routine.	0.513			
	Appreciating the landscape here makes me happy.	0.704			
	Appreciating the landscape here makes me feel energized.	0.743			
	Appreciating the landscape here relieves my sense of fatigue.	0.862			
	Appreciating the landscape here helps me concentrate better.	0.770			

Table 3 presents the means, standard deviations, and zero-order correlations among the five constructs; all construct means exceeded the scale midpoint (range: 3.13 to 4.02), and construal level showed the greatest variability (*SD* = 1.02), consistent with its theorized status as a situationally responsive cognitive variable. Discriminant validity was assessed via the Fornell–Larcker criterion (Fornell and Larcker, 1981). As shown in Table 3, the square root of each construct's AVE exceeded all of its correlations with other constructs (LE = 0.746; AW = 0.752; CL = 0.735; CN = 0.690; PRB = 0.743), confirming adequate discriminant validity across all five variables. Even for landscape extraordinariness and awe—the two constructs with the strongest theoretical overlap—both AVE square roots exceeded their mutual correlation (*r* = 0.532), statistically verifying the independence of these constructs.

**Table 3:** Means, standard deviations, correlations, and discriminant validity.

Construct	*M*	*SD*	LE	AW	CL	CN	PRB
**LE**	3.85	0.54	**0.746**				
**AW**	3.34	0.67	0.532	**0.752**			
**CL**	3.13	1.02	0.411	0.662	**0.735**		
**CN**	4.02	0.66	0.426	0.574	0.535	**0.690**	
**PRB**	3.66	0.78	0.256	0.124	0.170	0.362	**0.743**

Bold diagonal entries are square roots of AVE. LE = landscape extraordinariness; AW = awe; CL = construal level; CN = connectedness to nature; PRB = psychological restorative benefits.

### 4.3 Sub-sample comparability and measurement invariance

Before testing the structural model, we examined whether the two site-based sub-samples were sufficiently comparable for pooled analysis. Welch's *t*-tests showed no statistically significant differences between the Sanjiangyuan and Qinghai Lake sub-samples across the five core constructs, with all *p*-values above 0.05. The smallest *p*-value was observed for awe (*p* = 0.281), indicating that none of the construct-level differences approached statistical significance. Mann–Whitney U tests produced a similar non-significant pattern, with all *p*-values above 0.05, suggesting that the site-level comparison was not dependent on the assumptions of the parametric test.

Multi-group CFA was then conducted to examine measurement invariance across the two sub-samples. The configural model showed acceptable fit (CFI = 0.905, RMSEA = 0.048), indicating that the overall factor structure was comparable across the two sites. When factor loadings were constrained to be equal across groups, model fit remained stable (CFI = 0.904, RMSEA = 0.047; ΔCFI = 0.001), supporting metric invariance. The scalar model, in which measurement intercepts were further constrained, also showed no meaningful decline in fit (CFI = 0.903, RMSEA = 0.046; ΔCFI = 0.001). Together, these results suggest that the two sub-samples were sufficiently comparable in both observed construct scores and measurement structure, supporting their use as a pooled sample in the subsequent SEM analysis.

### 4.4 Structural model and hypothesis testing

**Figure 2:**
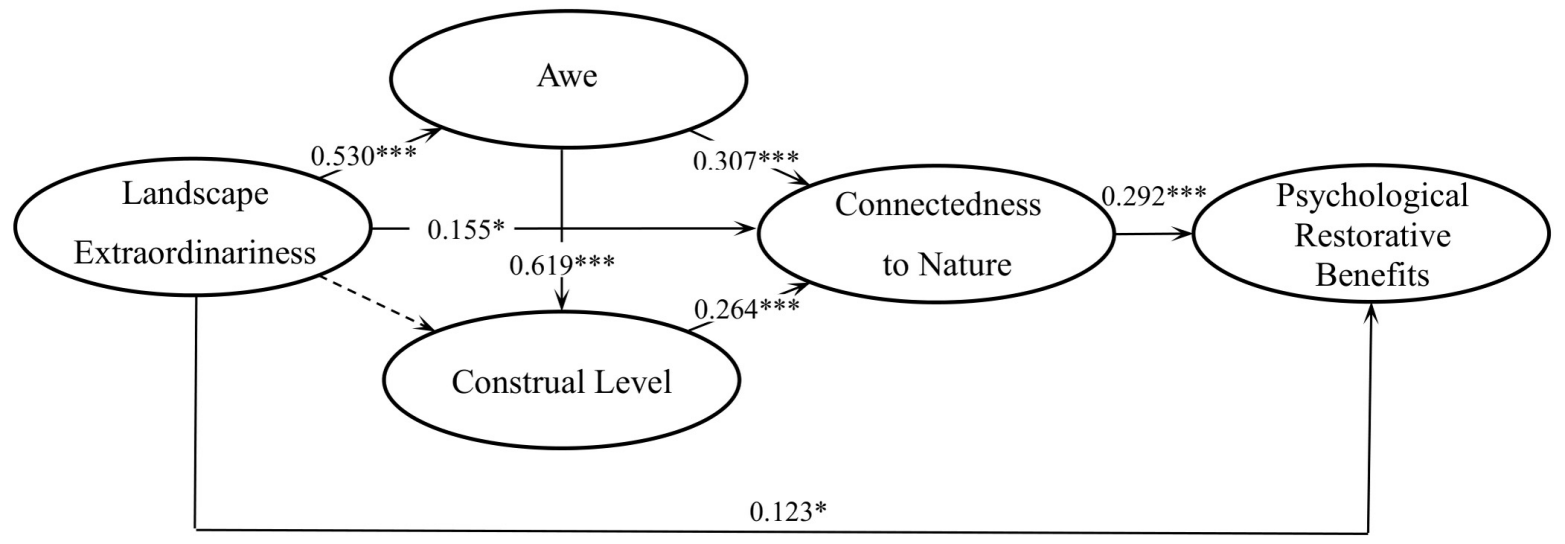
Standardized structural equation model results. *p < 0.05; ***p < 0.001.

The structural model showed adequate fit: χ^2^/ *df* = 3.105, RMSEA = 0.068, IFI = 0.904, TLI = 0.891, and CFI = 0.903. Standardized path coefficients are reported in Table 4 and Figure 2.

**Table 4:** Structural model results.

Hypothesis	Path	β	*SE*	*C.R*.	*p*	Supported
H1	LE → CN	0.155	0.096	2.563	< 0.05	Yes
H2a	LE → AW	0.530	0.143	9.105	< 0.001	Yes
H2b	AW → CN	0.307	0.051	3.863	< 0.001	Yes
H3a	LE → CL	0.082	0.158	1.419	0.156	No
H3b	CL → CN	0.264	0.046	3.385	< 0.001	Yes
H4a	AW → CL	0.619	0.070	9.779	< 0.001	Yes
H5	CN → PRB	0.292	0.050	4.645	< 0.001	Yes
H6	LE → PRB	0.123	0.074	2.118	< 0.05	Yes

Landscape extraordinariness exerted a significant positive direct effect on connectedness to nature (β = 0.155, *p* < 0.05), supporting H1. Landscape extraordinariness also significantly and positively predicted awe (β = 0.530, *p* < 0.001), supporting H2a; awe in turn significantly and positively predicted connectedness to nature (β = 0.307, *p* < 0.001), supporting H2b. The direct effect of landscape extraordinariness on construal level was non-significant (β = 0.082, *p* = 0.156), so H3a was not supported; however, higher-level construal was significantly associated with stronger connectedness to nature (β = 0.264, *p* < 0.001), supporting H3b. Awe was significantly associated with a higher level of construal (β = 0.619, *p* < 0.001), supporting H4a and supporting the proposed pathway within the hypothesized chain. Connectedness to nature positively and significantly predicted psychological restorative benefits (β = 0.292, *p* < 0.001), supporting H5. Landscape extraordinariness also exerted a significant direct effect on psychological restorative benefits (β = 0.123, *p* < 0.05), supporting H6. The model explained 28.1% of variance in awe, 38.3% in construal level, 49.6% in connectedness to nature, and 23.8% in psychological restorative benefits. All direct-effect hypotheses were supported with the exception of H3a.

### 4.5 Mediation analysis

Bias-corrected bootstrap analysis with 5,000 resamples and 95% confidence intervals was used to estimate the total, direct, and specific indirect effects of landscape extraordinariness on psychological restorative benefits. As shown in Table 5, the total effect estimate was 0.314 [95% CI (0.188, 0.462)]. The direct effect remained statistically significant after accounting for the mediators [estimate = 0.156, 95% CI (0.004, 0.311)], indicating partial mediation. The total indirect effect was also significant [estimate = 0.158, 95% CI (0.092, 0.250)].

**Table 5:** Mediation analysis results (Bootstrap, 5,000 resamples).

Effect type	Mediation path	Estimates	BootLLCI	BootULCI	*p*	Supported
Total effect	LE → PRB	0.314	0.188	0.462	< 0.001	Yes
Direct effect	LE → PRB	0.156	0.004	0.311	0.046	Yes
Total indirect effect	LE → PRB	0.158	0.092	0.250	< 0.001	Yes
Indirect 1	LE → CN → PRB	0.058	0.014	0.128	0.009	Yes
Indirect 2 (H2)	LE → AW → CN → PRB	0.060	0.025	0.114	0.001	Yes
Indirect 3 (H3)	LE → CL → CN → PRB	0.008	−0.002	0.029	0.099	No
Indirect 4 (H4b)	LE → AW → CL → CN → PRB	0.032	0.012	0.069	0.001	Yes

95% bias-corrected confidence intervals. BootLLCI/BootULCI = lower/upper confidence interval limits.

Among the specific indirect pathways, the indirect effect through connectedness to nature alone (LE → CN → PRB) was significant [estimate = 0.058, 95% CI (0.014, 0.128)]. The affective mediation path through awe and connectedness to nature (LE → AW → CN → PRB) was significant [estimate = 0.060, 95% CI (0.025, 0.114)], supporting H2. The independent cognitive mediation path through construal level and connectedness to nature (LE → CL → CN → PRB) was not significant [estimate = 0.008, 95% CI (−0.002, 0.029)]; therefore, H3 was not supported. The serial mediation path through awe, construal level, and connectedness to nature (LE → AW → CL → CN → PRB) was significant [estimate = 0.032, 95% CI (0.012, 0.069)], supporting H4b. Together, the indirect pathways specified in H2 and H4b accounted for 58.2% of the total indirect effect, with the affective pathway contributing the larger share.

### 4.6 Alternative model comparison

Two alternative structural models were compared with the proposed model to assess the relative consistency of different directional specifications with the observed data (Kline, 2016). Alternative Model 1 specified a cognitive-first sequence in which construal level preceded awe (LE → CL → AW → CN → PRB). Alternative Model 2 treated awe and construal level as parallel mediators and removed the directional path between them (LE → AW → CN → PRB and LE → CL → CN → PRB).

As shown in Table 6, the proposed affective-first model provided comparatively better fit on the reported indices. Compared with Alternative Model 1, the proposed model showed a lower AIC (1171.90 vs. 1220.49), a higher CFI (0.903 vs. 0.894), and a lower χ^2^/df value (3.105 vs. 3.282). Compared with Alternative Model 2, the proposed model also showed a lower AIC (1171.90 vs. 1186.77) and a higher CFI (0.903 vs. 0.899). The observed data were therefore more consistent with the proposed affective-first specification than with the cognitive-first or parallel specifications. However, model comparisons based on cross-sectional data do not establish temporal precedence.

**Table 6:** Alternative model comparison results.

Model	Specification	χ^2^/ *df*	CFI	TLI	IFI	RMSEA	AIC	BIC
Proposed model	LE → AW → CL → CN → PRB	3.105	0.903	0.891	0.904	0.068	1,171.9	1,437.26
Alternative model 1	LE → CL → AW → CN → PRB	3.282	0.894	0.879	0.896	0.071	1,220.49	1,485.85
Alternative model 2	Parallel (no AW–CL link)	3.173	0.899	0.886	0.901	0.069	1,186.77	1,444.82

## 5 Discussion

### 5.1 Discussion of findings

This study set out to address a notable gap in the nature–health literature: while the restorative potential of ordinary green spaces has been extensively documented, the psychological processes associated with exposure to extraordinary natural landscapes remain theoretically underdeveloped and empirically underexplored. Anchored in the Cognitive-Affective Personality System (CAPS) theory and hot/cool system theory, and situated in Sanjiangyuan National Park and Qinghai Lake National Park (candidate area), we conceptualized landscape extraordinariness as a continuous subjective perception variable and tested a chain mediation model linking it to psychological restorative benefits (PRB). Below, we discuss five substantive findings in turn.

First, landscape extraordinariness was strongly and positively associated with awe (β = 0.530, *p* < 0.001), and the affective mediation chain from landscape extraordinariness through awe and connectedness to nature to psychological restorative benefits yielded a significant indirect effect [estimate = 0.060, 95% CI (0.025, 0.114)], supporting an indirect pathway in which awe and connectedness to nature link extraordinary landscape perception with psychological restoration. This finding is consistent with Keltner and Haidt's (2003) prototypical model of awe, which proposes perceived vastness and the need for accommodation arising from schema incongruity as two core features of this emotion. It also extends the experimental findings of Bai et al. (2025) from laboratory-based image stimuli to an authentic national park setting, thereby providing field-based support for the relationship between extraordinary landscape perception and awe. Landscape extraordinariness also retained a significant direct association with connectedness to nature (β = 0.155, *p* < 0.05), indicating that awe did not fully account for the association between landscape perception and a sense of belonging to the natural world. The present model does not identify the processes underlying this remaining association. Future studies could examine perceived safety, threat appraisal, attentional processes, and other potential mediators by measuring them directly.

Second, the hypothesized direct path from landscape extraordinariness to construal level (H3a) was not supported (β = 0.082, *p* = 0.156), yet the downstream path from construal level to connectedness to nature was significant (H3b: β = 0.264, *p* < 0.001), and the serial mediation path from landscape extraordinariness through awe, construal level, and connectedness to nature to psychological restorative benefits was significant [H4b: estimate = 0.032, 95% CI (0.012, 0.069)]. The non-support of H3a, considered together with the significant chain mediation path, points to a potentially important boundary condition within the proposed model: the physical and spatial qualities of an extraordinary landscape may not, by themselves, be sufficient to support higher-level construal. Relatedly, Agrawal and Wan (2009) showed that construal level interacts with individuals' self-regulatory state in shaping information processing, suggesting that cognitive abstraction may depend on more than external stimulus properties alone. In the present context, the awe response—the emotional registration of the landscape's vastness and novelty—may facilitate a transition from concrete, detail-focused processing toward the abstract, meaning-oriented mode associated with higher construal level. The observed data pattern is consistent with the hot/cool system proposition that affectively salient, high-arousal contexts may preferentially engage the hot system before higher-order cool-system processing. However, this interpretation should be treated with caution, as the cross-sectional design cannot directly verify the temporal sequence of these psychological processes. If the proposed sequential order is supported by future longitudinal or experimental research, this finding may help explain why direct appeals to ecological significance or human–nature relationships could be less effective when presented before visitors have had sufficient opportunity to experience the landscape emotionally.

Third, connectedness to nature was positively associated with psychological restorative benefits (β = 0.292, *p* < 0.001), indicating that both the affective mediation path and the chain mediation path included this construct as the final mediator before psychological restorative benefits. This finding is consistent with Mayer et al. (2009) observation that connectedness to nature accounts for an important part of the relationship between nature exposure and psychological restoration, and it aligns with the biophilia hypothesis (Wilson, 1984), which attributes the health benefits of nature exposure to the fulfillment of an innate human need to affiliate with other living systems. Within the national park context, connectedness to nature appears to function as a proximal psychological link between visitors' affective engagement with extraordinary landscapes and their perceived restorative benefits, a pattern broadly consistent with the dual-process restorative model discussed by Bai et al. (2025). The bootstrap mediation results provide further quantitative support for this interpretation: both the affective path (LE → AW → CN → PRB) and the chain path (LE → AW → CL → CN → PRB) produced significant indirect effects, whereas the path without prior awe activation (LE → CL → CN → PRB) was not significant. Taken together, these findings highlight the central mediating role of connectedness to nature in the proposed model.

Fourth, landscape extraordinariness was also directly associated with psychological restorative benefits (β = 0.123, *p* < 0.05). The direct effect estimate [0.156, 95% CI (0.004, 0.311)] and the total indirect effect estimate [0.158, 95% CI (0.092, 0.250)] were similar in magnitude, indicating partial mediation. Taken together, the results are consistent with partial mediation. The present model does not identify the processes underlying the remaining direct association. Future studies could examine additional mediators and use longitudinal or experimental designs to assess their temporal relationships with psychological restorative benefits.

Fifth, the results support the validity of landscape extraordinariness as an independent, measurable construct distinct from awe. The five indicators—uniqueness (λ = 0.805), complexity (λ = 0.803), grandeur (λ = 0.803), remoteness (λ = 0.673), and mysteriousness (λ = 0.627)—together reflect the core perceptual attributes that distinguish extraordinary nature from its ordinary counterpart. Uniqueness, complexity, and grandeur showed the highest and nearly identical factor loadings, indicating that these attributes were strongly represented in visitors' overall evaluations of landscape extraordinariness. This pattern is consistent with Hao et al. (2024) emphasis on uniqueness as an important characteristic of extraordinary natural environments. Moreover, discriminant validity between landscape extraordinariness and awe was supported, indicating that the two constructs capture distinct psychological phenomena: the former reflects an appraisal of environmental properties, whereas the latter represents an affective response associated with those properties. By moving beyond the binary categorization of “ordinary vs. extraordinary nature” toward a continuous subjective perception variable, the measure provides a methodological basis for comparative research across different extraordinary landscape contexts.

### 5.2 Theoretical implications

This study makes several contributions to the literature on extraordinary nature, awe, and the restorative effects of natural environments. First, it advances the conceptualization and operationalization of extraordinary nature by treating landscape extraordinariness as a continuous subjective perception variable. Prior research has commonly treated extraordinary nature as a dichotomous environmental category (Joye and Bolderdijk, 2015), which limits the examination of individual variation in perceived extraordinariness. By adapting and evaluating a five-item semantic differential measure in national park settings, the present study provides an initial measurement basis for examining variations in landscape extraordinariness across visitors and environmental contexts.

Second, this study integrates CAPS theory, hot/cool system theory, and Construal Level Theory to specify an affective–cognitive pathway linking landscape extraordinariness with psychological restorative benefits. Existing nature–health research has often examined affective and cognitive variables as parallel or independent mediators (Bratman et al., 2015; Collado and Manrique, 2020). In the present study, the proposed affective-first model provided comparatively better fit than the cognitive-first and parallel specifications, and the serial indirect path through awe, construal level, and connectedness to nature was significant. These findings are consistent with a model in which awe is positioned before higher-level construal and connectedness to nature in the proposed pathway. By extending the serial indirect association to psychological restorative benefits, the study connects affective and cognitive processing with both nature-related identification and perceived restoration.

Third, the study extends extraordinary nature research by examining the proposed associations in national park settings characterized by large-scale and distinctive landscapes. Whether management conditions, perceived safety, or threat appraisal shape these associations remains an open question for comparative research across differently managed settings.

### 5.3 Practical implications

The positive association between landscape extraordinariness and awe has implications for national park planning and visitor experience design. Park managers may prioritize the preservation and presentation of landscape attributes associated with uniqueness, grandeur, and ecological complexity. Practical measures could include locating viewpoints and trails to frame expansive vistas, protecting biodiversity-rich areas that contribute to perceived complexity, and developing interpretive materials that communicate the ecological rarity and distinctive character of the landscape. Management efforts should also avoid excessive homogenization of visitor infrastructure that may reduce the perceptual distinctiveness of key landscape settings. Park authorities could conduct periodic landscape-experience assessments in which first-time visitors evaluate the perceived uniqueness, grandeur, and complexity of major viewpoints before substantial infrastructure changes are approved.

The significant serial indirect effect suggests that the sequencing of interpretive programming may be a useful topic for experimental evaluation. Park managers could pilot-test alternative sequences rather than assuming that one sequence is universally optimal. For example, one visitor group could receive ecological and human–nature relationship content at the entrance, while another group could receive the same content after reaching a major viewpoint or mid-trail rest station. Differences in awe, construal level, connectedness to nature, and perceived restoration could then be compared across the two conditions. Designated rest stations could also display QR codes that visitors may voluntarily scan to access brief reflective audio content concerning ecological interdependence, conservation values, or the geological history of the landscape. Such trials could help determine whether the timing of interpretive content influences visitors' psychological responses and restorative experiences.

From a public health perspective, the findings may inform the development of nature-based wellbeing initiatives in national park settings. National park managers and community health organizations could explore pilot partnerships that provide structured park experiences for populations experiencing high levels of stress or limited access to extraordinary natural environments. Such programs might combine landscape access with brief interpretive or reflective activities designed around awe and connectedness to nature. Pilot programs should evaluate participation, accessibility, equity, psychological outcomes, and cost-effectiveness before being expanded or positioned as complements to conventional mental health services. One possible pilot model would involve subsidized park visits for high-stress occupational groups, accompanied by a brief pre-visit orientation and a post-visit reflection activity.

## 4 Limitations and future research

This study has several limitations that point to directions for future research. First, both field sites are located on the Qinghai–Tibet Plateau and represent a relatively narrow range of extraordinary landscapes, including high-altitude grasslands, glacial lakes, and canyon environments. In addition, the convenience sampling design limits population representativeness. Further validation is therefore needed across different landscape types, cultural settings, and visitor populations to determine the broader applicability of both the measurement instrument and the structural model. Second, the cross-sectional design captures visitors' perceptions at a single point in time and therefore cannot establish causal relationships or directly verify the proposed temporal ordering of awe and construal level. Although the proposed model provided comparatively better fit than the cognitive-first and parallel alternatives, these model comparisons cannot establish temporal precedence. Longitudinal or experimental designs with temporally separated measurements could provide stronger evidence concerning the proposed sequence. Third, all constructs were measured using self-report instruments. Future studies could combine self-reported restoration with physiological or behavioral indicators, such as cortisol reactivity, heart-rate variability, or post-visit attentional performance. Fourth, the study did not include measures of perceived safety, environmental threat, attentional processes, attentional recovery, or physiological stress responses. Future studies could include these variables to examine whether they help account for the remaining direct associations between landscape extraordinariness, connectedness to nature, and psychological restorative benefits. Finally, the present study examined a limited set of affective and cognitive mediators. Future research could compare connectedness to nature with other potential explanatory variables, including positive affect, self-transcendence, perceived escape, and attentional restoration.

## Data Availability

The raw data supporting the conclusions of this article will be made available by the authors, without undue reservation.
